# A possible role for taste receptor cells in surveying the oral microbiome

**DOI:** 10.1371/journal.pbio.3001953

**Published:** 2023-01-13

**Authors:** Emma M. Heisey, Lynnette Phillips McCluskey

**Affiliations:** 1 PhD Program in Neuroscience, Medical College of Georgia at Augusta University, Augusta, Georgia, United States of America; 2 Department of Neuroscience & Regenerative Medicine, Medical College of Georgia at Augusta University, Augusta, Georgia, United States of America

## Abstract

Taste receptor cells are sensory specialists that detect chemicals in food and drink. An exciting new report in *PLOS Biology* suggests that some taste cells could also be involved in immune surveillance like counterparts in the intestine.

Taste receptor cells on the tongue are known for sensing chemical stimuli that allow us to enjoy or reject food. The sense of taste affects quality of life and nutrition as experienced by many afflicted by taste loss from cancer treatments or Coronavirus Disease 2019 (COVID-19) infection. Animals sample the environment to discriminate nutritive, often sweet foods from bitter toxins. Yet oral taste buds, composed of a heterogeneous population of taste cells, are also exposed to a diverse array of microbes second only to the gut microbiome. It’s become clear that healthy and injured taste cells on the tongue communicate with the immune system. For example, taste buds express pathogen-detecting toll-like receptors, cytokines, and their receptors and respond to cytokines by altering sensory responses and modulating taste cell turnover [1–3]. An article by Qin and colleagues in *PLOS Biology* offers intriguing evidence that sweet- and umami-sensing taste cells in mice may also surveil the oral microbial environment [4].

Single-cell RNASeq analyses revealed that in fact, some taste cells share a gene expression profile similar to microfold (M) cells in the intestine. M cells sample the intestinal microbiome by transporting antigens from the lumen to underlying immune cells, triggering either mucosal immune responses or tolerance (Fig 1A and 1B). Taste receptor cells expressing *Tas1r3* gene are a subset of type II cells bearing G-protein coupled receptors that recognize sweet and umami stimuli. A distinct pool of type II cells respond to bitter, while type I cells are glial-like and type III cells sense sour and salty tastants [5]. Compared to surrounding non-taste epithelium, *Tas1r3*+ cells express higher levels of M cell markers, including *Spib*, which encodes a critical transcription factor in M cell development. The team from the Sukamaran laboratory used RNAscope and immunohistochemistry to confirm the identity of 95% of *Spib*+ taste cells as *T1r3+* type II taste cells (Fig 1B).

**Fig 1:**
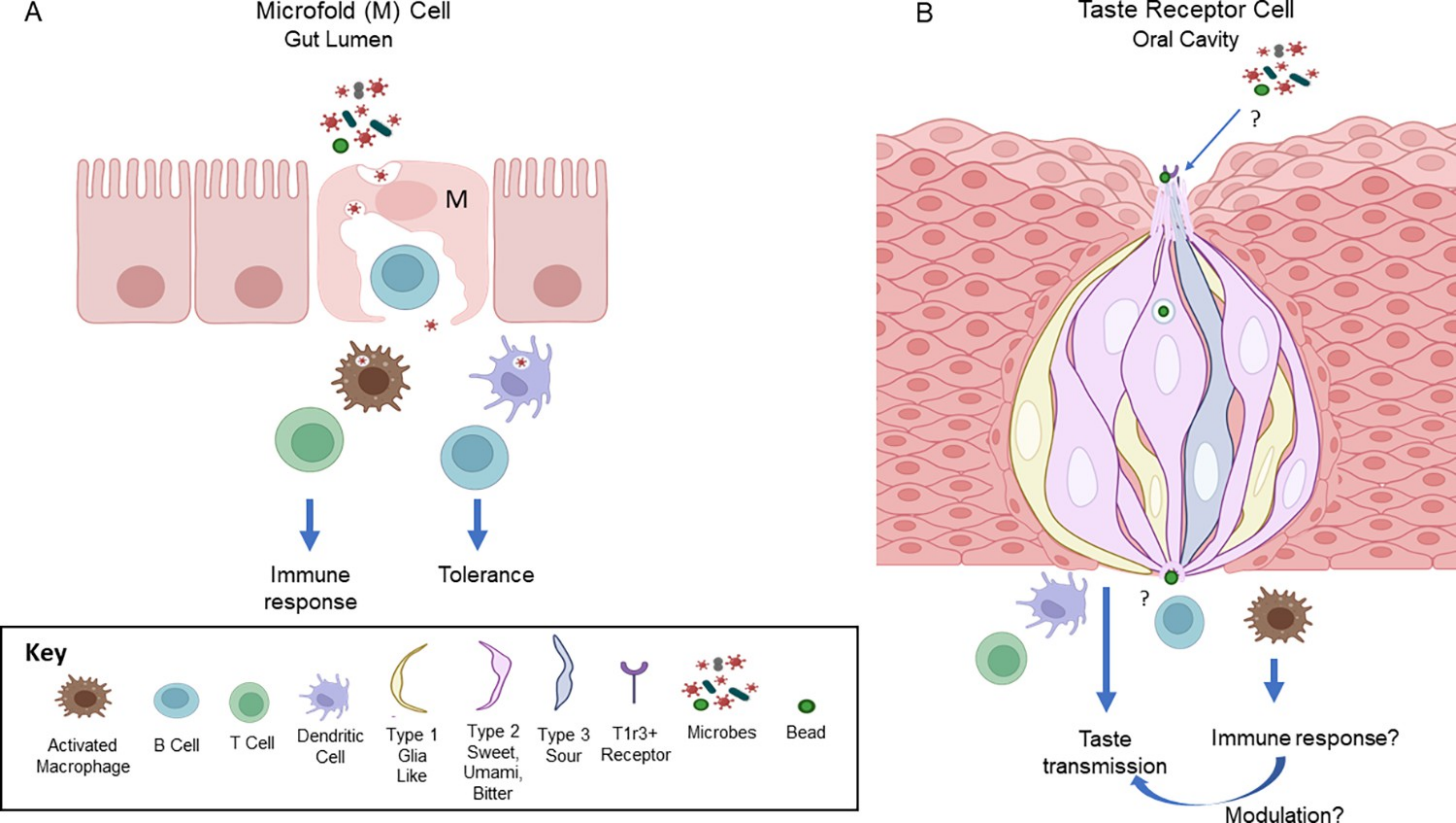
Microfold (M) cell surveillance of the microbiota and a similar potential role for taste cells on the tongue. **(A)** The gut microbiota is sampled by M cells that deliver bacteria and viruses across the epithelial layer to underlying immune cells, triggering immune responses or tolerance in the mucosa. The M cell pocket is occupied by a B lymphocyte that directs the maturation of M cell function. **(B)** Heterogeneous groups of taste cells on the tongue form taste buds surrounded by epithelial cells. Unlike M cells, taste cells are tipped by microvilli projecting into the oral cavity. A new study by Qin and colleagues demonstrates that sweet- and umami-sensing *T1r3*+ cells also express signature M cell genes such as the transcription factor, *Spib*. Wild-type taste buds take up fluorescent latex beads similar to M cells but uptake is reduced in *Spib* KO taste buds. Immune cell density under taste buds is also diminished in the absence of *Spib*. Whether taste cells can transport beads or microbes to immune cells and trigger immune responses remains to be tested. Intriguingly, *Spib* KO mice had increased licking responses to sucrose and umami tastants though the significance of taste changes is unknown. This exciting new study indicates a novel role of some taste cells in sampling the oral microbiome in homeostasis and during infection. This figure was created with BioRender.com.

*Spib* KO mice were key in exploring functional similarities between taste and M cells. RANKL, a member of the TNF ligand superfamily, induces intestinal M cell development and might act similarly on taste cells [3]. Indeed, RANKL increased the proportion of taste cells expressing M cell markers and up-regulated these genes in wild type but not *Spib* KO taste buds and taste organoids. The authors conclude that RANKL stimulated the proliferation of M-like taste cells, though that remains to be demonstrated directly. Together, these experiments provide evidence that a specific subset of taste cells expresses M cell markers, but can they also modulate immune responses? The answer is yes, as many genes in the NFκB pathway and downstream cytokines are down-regulated in taste tissue from *Spib* KO mice. Importantly, baseline immune responses are drastically reduced in *Spib* KO taste tissue. Immune cells including macrophages, neutrophils, and T cells were prominent in the tissue underlying taste buds in wild-type mice but minimal in the absence of *Spib*.

Classical M cells overlay well-organized immune centers known as mucosa-associated lymphoid tissue (MALT). M cells transport microbes across the mucosal barrier and deliver them to immune cells like dendritic cells in a process known as transcytosis [3]. The localization of immune cells beneath *T1r3+* taste cells is reminiscent of MALT though true lymphoid tissue is located at the base of the tongue in humans. To begin to assess pathogen surveillance in M-like taste cells, the authors applied fluorescent latex beads to the tongue and measured their uptake by taste buds (Fig 1B). Beads were contained within taste buds from control but not *Spib* KO mice, providing new evidence that taste cells potentially sample the oral cavity. Caveats are that to demonstrate transcytosis the beads should appear on the basal side of taste buds with time, presumably in immune cells that carry out the next steps in handling pathogenic threats. Confirming that *T1r3+* taste cells are delivering the cargo is another next step since the authors used general taste markers. However, this initial evidence that taste cells function in immune surveillance is novel and will stimulate further studies.

While a specific subset of taste cells express M cell markers and modulate immune responses, the authors tested if this subset could also regulate taste behavior. Gustometers were used to deliver small volumes of taste solutions and measure *Spib* KO animals’ responses by calculating the number of licks to tastants divided by licks to water. Lick ratios that are >1 indicate enhanced preference that was reported in response to the sweet (sucrose) and umami (MPG) tastants, but not to sucralose, a nonnutritive sweetener or bitter and sour tastants. There is currently no explanation for enhanced preference for sucrose and MPG but not sucralose since this experiment tests taste behaviour and not nutritive quality. Curiously, the enhanced responses are not explained by a change in the number of taste receptor cells. The mechanism, therefore, remains elusive and open to further exploration.

Like most novel findings, this study raises a number of questions. How could microvilli-tipped taste cells be specialized to interact with microbes? M cells are thought to bind rather than repel bacteria because they lack microvilli, appearing as dimples in the intestinal barrier [3]. The major open question is whether taste cells are truly sampling the microbial environment triggering mucosal immune responses. The specific steps in pathogen sampling by *T1r3+* cells might differ from the intestine but it will be critical to test immunological outcomes and their effects on taste responses. A definitive role for taste cells in immune surveillance could reveal mechanisms underlying taste loss in conditions that alter the oral microbiome. Tongue swabs revealed a higher abundance of microbiota that cause inflammation in patients with long COVID as well as chronic fatigue syndrome, though further studies with larger sample sizes are needed to link taste deficits [6]. Overall, Qin and colleagues have provided a glimpse at a potential new role for taste cells as microbiota sensors in health and disease.

## References

[1] Lakshmanan HG, Miller E, White-Canale A, McCluskey LP. Immune responses in the injured olfactory and gustatory systems: a role in olfactory receptor neuron and taste bud regeneration? *Chem Senses*. 2022;47. doi: 10.1093/chemse/bjac024 36152297 PMC9508897

[2] Wang H, Zhou M, Brand J, Huang L. Inflammation and taste disorders: mechanisms in taste buds. Ann N Y Acad Sci. 2009;1170:596–603. doi: 10.1111/j.1749-6632.2009.04480.x 19686199 PMC2729510

[3] Dillon A, Lo DD. M Cells: Intelligent Engineering of Mucosal Immune Surveillance. Front Immunol. 2019;10:1499. doi: 10.3389/fimmu.2019.01499 31312204 PMC6614372

[4] Qin Y, Palayyan SR, Zheng X, Tian S, Margolskee RF and Sukumaran SK. Type II taste cells may participate in mucosal surveilliance. PLoS Biol. 2023; 21(1):e3001647. doi: 10.1371/journal.pbio.3001647 36634039 PMC9836272

[5] Roper SD, Chaudhari N. Taste buds: cells, signals and synapses. Nat Rev Neurosci. 2017;18(8):485–497. doi: 10.1038/nrn.2017.68 28655883 PMC5958546

[6] Haran JP, Bradley E, Zeamer AL, Cincotta L, Salive M-C, Dutta P, et al. Inflammation-type dysbiosis of the oral microbiome associates with the duration of COVID-19 symptoms and long COVID. JCI Insight. 2021;6(20). doi: 10.1172/jci.insight.152346 34403368 PMC8564890

